# Supplementary material for: Establishing In-House Cutoffs of CSF Alzheimer’s Disease Biomarkers for the AT(N) Stratification of the Alzheimer Center Barcelona Cohort
Source: Int J Mol Sci. 2022 Jun 21;23(13):6891. doi: 10.3390/ijms23136891 (PMC9266894; doi:10.3390/ijms23136891)
Supplement: Supplementary file 1 [file ijms-23-06891-s001.zip › Supplemental Figures.pptx]

## Slide 1
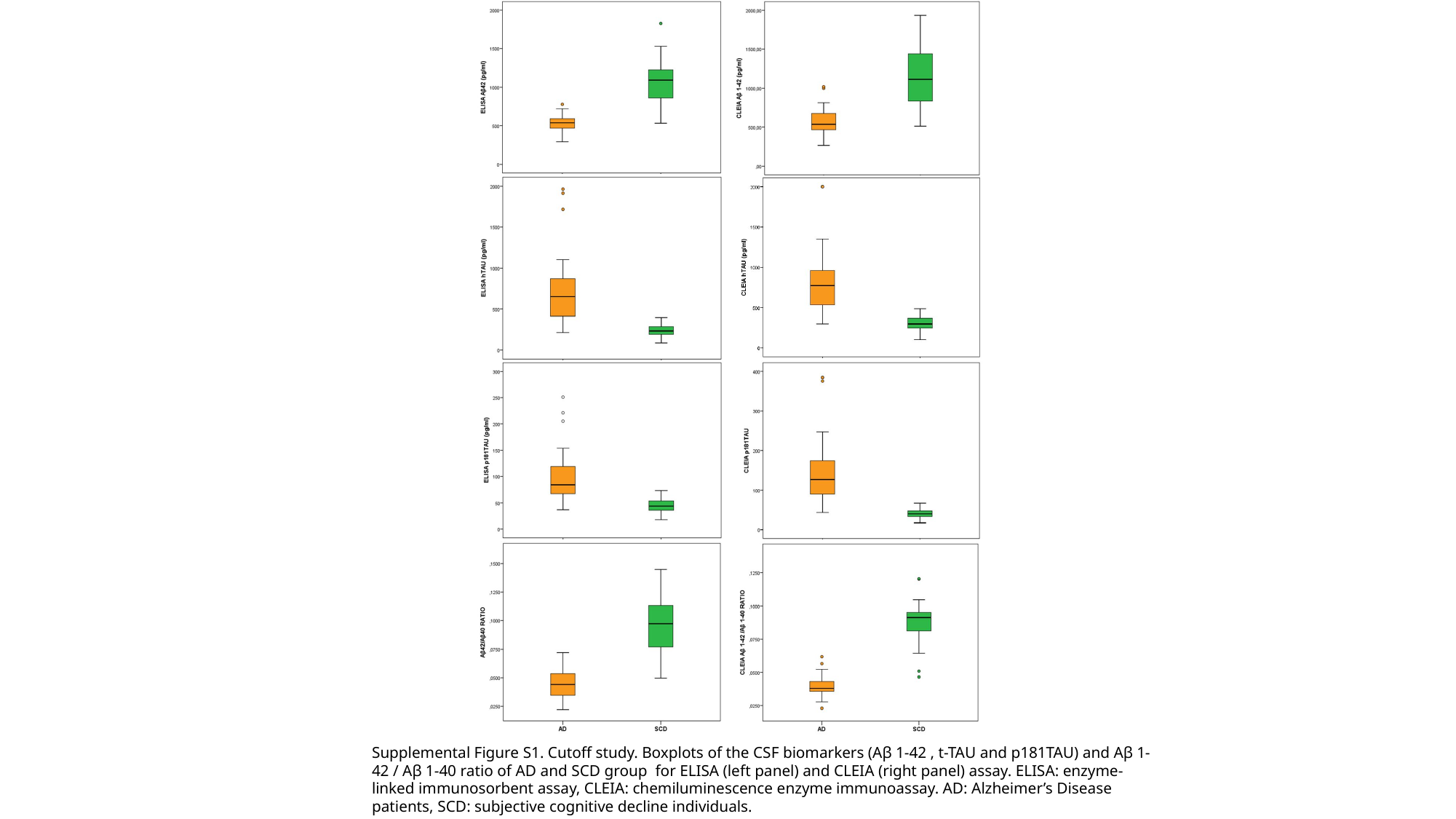

Supplemental Figure S1. Cutoff study. Boxplots of the CSF biomarkers (Aβ 1-42 , t-TAU and p181TAU) and Aβ 1-42 / Aβ 1-40 ratio of AD and SCD group for ELISA (left panel) and CLEIA (right panel) assay. ELISA: enzyme-linked immunosorbent assay, CLEIA: chemiluminescence enzyme immunoassay. AD: Alzheimer’s Disease patients, SCD: subjective cognitive decline individuals.

## Slide 2
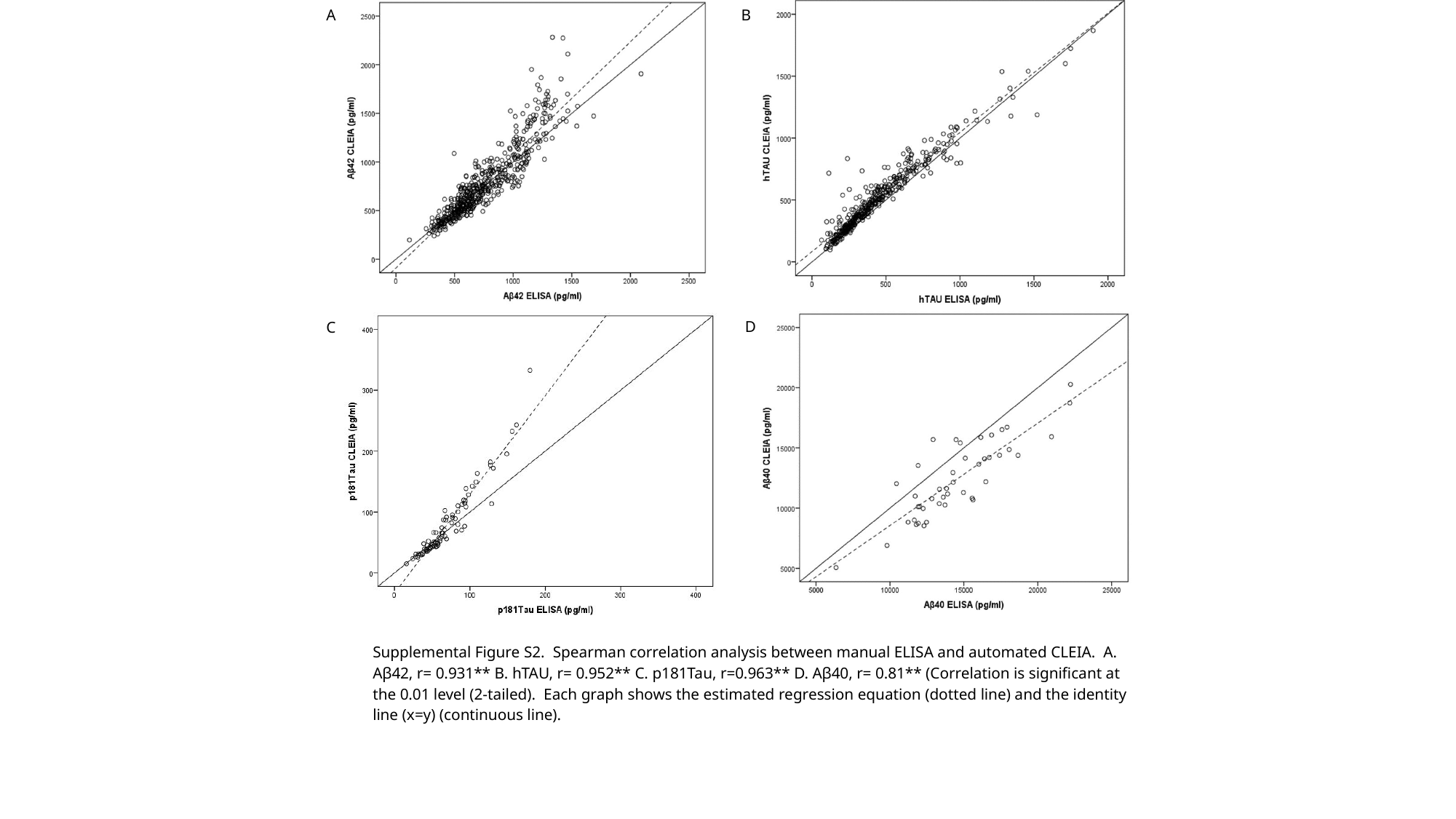

A
B
D
C
Supplemental Figure S2. Spearman correlation analysis between manual ELISA and automated CLEIA. A. Aβ42, r= 0.931** B. hTAU, r= 0.952** C. p181Tau, r=0.963** D. Aβ40, r= 0.81** (Correlation is significant at the 0.01 level (2-tailed). Each graph shows the estimated regression equation (dotted line) and the identity line (x=y) (continuous line).
