# Supplementary material for: Establishing In-House Cutoffs of CSF Alzheimer’s Disease Biomarkers for the AT(N) Stratification of the Alzheimer Center Barcelona Cohort
Source: Int J Mol Sci. 2022 Jun 21;23(13):6891. doi: 10.3390/ijms23136891 (PMC9266894; doi:10.3390/ijms23136891)
Supplement: Supplementary file 1 [file ijms-23-06891-s001.zip › Supplemental Methods.pdf]

SUPPLEMENTAL METHODS

CSF and plasma collection protocol

Lumbar Puncture (LP) and CSF collection

The lumbar puncture (LP) was performed by an experienced neurologist with the patients in sitting position and under fasting conditions. After applying subcutaneously local anesthesia (1% mepivacaine), CSF was obtained by LP in the intervertebral space of L3-L4. The fluid was collected passively in two 12-ml polypropylene tubes (Sarstedt Ref 62.610.018). The first tube of CSF was analyzed externally for basic biochemistry (glucose, total proteins, proteinogram, cell type and cell number). The second tube is centrifuge (2000xg 10 min at 4ºC) and variable volumes of the fluid is aliquot into polypropylene tubes (Sarstedt Ref 72.694.007) and stored at -80ºC until used. The time delay between CSF collection and storage was less than 2 hours.

Plasma and Serum collection

Consecutively to the CSF matched samples of serum and plasma were obtained from the patient. Briefly, blood was collected in BD Vacutainer tubes. Three of them (1x SSTII Advance and 2x K2-EDTA, respectively) were centrifuge (2000xg, 10mín at 4°C) to obtain serum and plasma. To obtain the white cells fraction, pellets from plasma tubes were processed by lysis buffer (20 mM Tris pH7.5, 5 mM MgCl2), soft shaking and centrifugation (4000xg, 15min, 4°C).Supernatants were aliquoted and stored at -80°C. Two other tubes were analyzed externally: one (K3-EDTA) for the blood cell count and the other (SSTII Advance) for basic analysis (glucose, total proteins, proteinogram).

APOE genotyping

Genomic DNA was obtained from frozen whole blood collected in BD Vacutainer tubes (K2-EDTA). DNA extraction was performed using *C*hemagen platform (Perkin Elmer). Afterwards, *APOE* genotype was determined by TaqMan probes analysis of SNP ID RS429358 and SNP ID RS7412 using a Real-Time PCR system QuantStudio3 (Thermofisher).

Ethical Considerations

All the samples collected have written consent of the subjects participating in the study. These consent protocols have been previously approved by Ethical Committee of the Hospital Clínic I Provincial de Barcelona (HCB/2014/0494, HCB/2016/0571, HCB/2016/0835, HCB/2017/0125 and HCB/2018/0333). The protocols have been designed in agreement with the indications of the *Sociedad Española de Neurologia (www.sen.es)* according to the current regulations for the use of clinical data and biological material and surplus of the assisted process for the biomedical research of neurodegenerative diseases.

FACEHBI participants study gave additional written consent, and its protocol was approved by the ethics committee of the Hospital Clinic i Provincial (Barcelona, Spain) (EudraCT: 2014-000798-38).
