# Supplementary material for: Establishing In-House Cutoffs of CSF Alzheimer’s Disease Biomarkers for the AT(N) Stratification of the Alzheimer Center Barcelona Cohort
Source: Int J Mol Sci. 2022 Jun 21;23(13):6891. doi: 10.3390/ijms23136891 (PMC9266894; doi:10.3390/ijms23136891)
Supplement: Supplementary file 1 [file ijms-23-06891-s001.zip › Supplemental Tables.pdf]

Supplemental Table S1. Study 1 SCD participants of FACEHBI study excluded as controls. Participants of the FACEHBI that underwent LP (n=55).Of them, n=2 had PET positive (SUVR>1.45) and were clinically diagnosed as MCI, n=6 had PET positive and n=2 were clinically diagnosed as MCI. The average time window between LP and FBB-PET evaluations was 1.3 ±1.4 months (range -1.68 to 5.29 months).

SCD excluded (n)	FBB-PET (V2)	MCI Clinical Diagnosis
2	+	+
6	+	-
2	-	+

SCD: subjective cognitive decline, FACEHBI: Fundació ACE Health Brain Study, LP: Lumbar Puncture, MCI: mild cognitive impairment, FBB-PET: 18F-Florbetaben-labeled positron emission tomography (FBB-PET), SUVR: FBB-PET global standardized uptake value ratio.

Supplemental Table S2. Study 2 Comparison of Immunoassay. Description statistics of CSF biomarkers concentrations obtained by ELISA and CLEIA methods in the correlation study.

Assay	N	Mean	Median	Std. Deviation	Minimum	Maximum
Aβ1-42 ELISA	519	743	676	272	115	1426
Aβ1-42 CLEIA[a]	519	776	697	356	197	2282
hTAU ELISA	364	414	376	203	94	968
hTAU CLEIA[a]	364	477	439	222	97	1087
P181TAU ELISA	77	70	63	34	16	180
P181TAU CLEIA[b]	77	81	60	57	15	332
Aβ1-40 ELISA	44	14499	14083	3182	6354	22220
Aβ1-40 CLEIA[b]	44	12386	11833	3192	5066	20284

CSF: cerebrospinal fluid, ELISA: enzyme-linked immunosorbent assay, CLEIA: chemiluminescence enzyme immunoassay. Description Statistics were given in pg/ml. [a]Launched by Fujirebio in April 2017. [b]Launched by Fujirebio in October 2018

Supplemental Table S3. Study 2 Comparison of Immunoassays. Summary of Passing-Bablok regression analysis of automated CLEIA and manual ELISA

Biomarkers	n	Slope	95%IC slope	Intercept	95%IC intercept
Aβ1-42	519	1.20	1.15 to 1.26	-126.5	-159.6 to -92.72
hTAU	364	1.12	1.09 to 1.14	10.52	1.09 to 20.4
p181TAU	77	1.54	1.42 to 1.66	-28.48	-35.33 to -19.63
Aβ1-40	44	1.03	0.85 to 1.22	-2707.21	-5338.50 to -233.23

ELISA: enzyme-linked immunosorbent assay, CLEIA: chemiluminescence enzyme immunoassay. Regression analysis data for all biomarkers were given in pg/ml

Supplemental Table S4: Study 3 Concordance between immunoassays. Demographic, clinical, genetic and biomarker data measured with manual ELISA and automated CLEIA.

	All	MCI	Dementia	χ2/T test /U	p-value
n (%)	68(100%)	48 (71%)	20 (29%)		
Age, years	72.4 (7.5)	70.8 (7.29)	78.3 (6.6)	2.89	0.005
Gender, Female/Male (% Female)	37/58(54%)	24/24 (50%)	13/7 (65%)	0.75	0.387
APOEε4 +/- (%+) (n=67)	25/42(37%)	17/31 (35%)	8/11 (42%)	0.05	0.818
MMSE score	24..4 (3.8)	26.2(2.7)	20.2 (2.8)	8.30	<0.0001
**CSF biomarkers, ELISA**					
Aβ1-42	786 ±308	816 ±336	717±337	382.50	0.189
Aβ1-40	14033±3749	13724±3625	14775±4029	390.00	0.226
t-Tau	396±321	313±145	594±502	316.50	0.028
p181Tau	63 ±33	56±22	81±46	322.50	0.034
Aβ1-42/Aβ1-40	0.058±0.023	0.062±0.022	0.051±0.021	390.00	0.053
**CSF biomarkers, CLEIA**					
Aβ1-42	838±397	875±422	747±321	406.00	0.319
Aβ1-40	11945±3519	11632±3508	12697±3519	378.00	0.170
t-Tau	496±325	418±189	685±481	325.00	0.037
p181Tau	74±56	61±34	105±83	305.00	0.018
Aβ1-42/Aβ1-40	0.071±0.027	0.076±0.026	0.061±0.025	303.00	0.017

MCI: Mild Cognitive Impairment, CSF: cerebrospinal fluid, APOE ε4: apolipotrein allele e4 carriers, MMSE: mini-mental state examination. Data are presented as mean (SD) otherwise specified. p values were calculated by comparing MCI and Dementia patients using Student_T-test, χ2 and Mann-Whitney U . All biomarkers data were presented as pg/ml.

Supplemental Table S5. Cutoff comparison of CLEIA[a] assay

Biomarkers	Source	AUC(95%IC)	Cutoff[b]	Youden J Index	Sensitivity	Specificity
Aβ42						
	FACE	0.927 (0.875-0.980)	<796	0.72	92	80
	Fujirebio	0.833 (0.747-0.918)	<599	0.597	78	81
	Bayart *et al*[37]	0.79 (0.680-0.890)	<381	NA	77.3	81
	Leitão *et al*	0.858 (0.783-0.933)	<543	NA	85	78
t-Tau						
	FACE	0.952 (0.905-0.999)	>412	0.81	92	89
	Fujirebio	0.922 (0.858-0.922)	>404	0.857	95	91
	Bayart *et al*[37]	0.9 (0.83-0.96)	>381	NA	86	83
	Leitão *et al*[38]	0.968 (0.949-0.997)	>335	NA	91	92
p181Tau						
	FACE	0.97 (0.939-1.000)	>54	0.83	92	91
	Fujirebio	0.995 (0.986-1.003)	>56.5	0.967	97	100
	Leitão, *et al*[38]	0.986 (0.969-1.000)	>50.6	NA	96	95
Aβ42/ Aβ40						
	FACE	0.993(0.982.1.000)	<0.063	0.95	100	95
	Fujirebio	0.976(0.947.1.004)	<0.069	0.89	98	91
	Leitão, *et al*[38]	0.960(0.960-1.000)	<0.068	NA	97.5	92.5

ROC: receiver operating characteristic, AUC: area under the curve, CSF: cerebrospinal fluid, [a]CLEIA: chemluminescence enzyme immunoassay. [b]Cutoff for single biomarkers are given in pg/ml. NA: not available. FACE: Fundació ACE

Supplemental Table S6. RHO spearman coefficients among statistically signifcant variables associated to MCI to Dementia phenoconversion

	ATN categories	AGE	MMSE	MCI categories
ATN Categories	1	,273**	-,193**	,287**
AGE	,273**	1	-,321**	,301**
Baseline MSE	-,193**	-,321**	1	-,339**
MCI categories	,287**	,301**	-,339**	1

** p<0.01
